# Advantages of ultrasound in the emergency room in a septic shock patient

**DOI:** 10.1186/2036-7902-7-S1-A17

**Published:** 2015-03-09

**Authors:** M Algaba-Montes, A Oviedo-García

**Affiliations:** 1 Emergency Department, Valme Hospital, Seville, Spain

## Background

Pyonephrosis (PN) is an uncommon disease that is associated with suppurative destruction of the renal parenchyma in adults. Obstruction and upper urinary tract infection play a role in its etiology. Fever, shivering, and flank pain are frequent clinical symptoms. If the pus detected as a result of the investigations is not surgically drained, antibiotics may not be effective. Septic shock and death can occur if the disorder is not treated with urgent surgery. In this context percutaneous or open nephrostomy or retrograde ureteral catheter insertion is appropriate, so it is a very serious disease and EP have a very important role in early diagnosis to start antibiotic treatment and early referral to surgery.

## Objective

We present a case of septic condition with PN developing due to a distal ureteral stone, diagnosed at ER, through the use of US scanning used by EP

## Patients and methods

A patient with fever, right flank pain and septic condition, with a final diagnosis of a calculous PN.

## Results

A 56 year old male, was admitted to the ER by right flank pain and fever. On arrival had malaise, hypotensive, febrile, tachycardic... in septic shock condition. Bedside emergency abdominal US was performed by EP, demonstrating right moderate to severe pelvocaliectasis due to a distal right ureteral stone. The patient was started on empirical antibiotics and a retrograde ureteral internal stents was placed by urologist.

## Conclusion

Identifying PN with early obstructive uropathy is clinically important in the ED because obstructive urolithiasis is an independent risk factor for inpatient death; so PN is a life-threatening condition. Emergent bedside ultrasound can do that EP may dramatically increase their ability to identify those patients that need further investigation, consultation and ultimately increase patient safety in emergency department. In the case presented thanks to the implementation of emergency US by the EP came to a prompt diagnosis of the cause of septic shock, with a quickly drainage of the infection site, which it allowed rapid patient recovery.

## Informed consent

The study was conducted in accordance with the ethical standards dictated by applicable law. Informed consent was obtained from each owner to enrolment in the study and to the inclusion in this article of information that could potentially lead to their identification.
